# Charles A. Wall, Jr.

**Published:** 1917-09

**Authors:** 


**Charles A. Wall, Jr.,** son of Dr. Charles A. Wall of Buffalo, having qualified as an aviator for the U. S. Army and expecting soon to be sent to France, was burned to death while experimenting in an aeroplane with a new bomb, at the Curtiss Aviation Field, Aug. 20.

